# Advances in understanding the role of mitochondria in renal ischemia–reperfusion injury

**DOI:** 10.1007/s10157-025-02727-3

**Published:** 2025-07-08

**Authors:** Huimeng Li, Xiangbo Wang, Danfang Deng, Shenhui Lv, Lili Huang, Xiaoqin Wang

**Affiliations:** 1 Hubei University of Chinese Medicine, Wuhan, 430061 China; 2 Hubei Provincial Hospital of Traditional Chinese Medicine, Wuhan, 430061 China; 3 Hubei Key Laboratory of Theory and Application Research of Liver and Kidney in Traditional Chinese Medicine, Affiliated Hospital of Hubei University of Chinese Medicine, Wuhan, 460061 China; 4 Hubei Shizhen Laboratory, Wuhan, 430061 China; 5 Department of Nephrology, Hubei Provincial Hospital of Traditional Chinese Medicine, 856 Luoyu Road, Hongshan District, Wuhan, 430074 Hubei China

**Keywords:** Autophagy, Biogenesis, Dynamics, Mitochondria, Renal ischemia–reperfusion injury

## Abstract

Renal ischemia–reperfusion injury (RIRI) is a major cause of acute kidney failure. Recent studies have shown that RIRI mechanism is closely related to abnormal mitochondrial biogenesis, fusion, fission, and autophagy. Maintaining normal mitochondrial function is essential for RIRI treatment. Therefore, it is important to explore molecular mechanisms of RIRI and relevant therapeutic targets. This review describes the role of mitochondria in RIRI and summarises information about potential drugs that regulate mitochondrial function, with the aim of providing ideas for clinical targeting of mitochondria to prevent and treat RIRI.

## Introduction

Renal ischaemia–reperfusion injury (RIRI), caused by the initial disruption and subsequent recovery of renal blood supply, is an important cause of acute kidney injury (AKI) [1], which mainly occurs in patients with shock, renal vascular occlusion, renal transplantation, and those undergoing cardiac surgery. RIRI is relatively prevalent, has a poor prognosis, lacks effective means of prevention and treatment, and represents a global public health problem [2].

Mitochondria are semi-autonomous organelles with their own unique genome that function as energy and signalling hubs, being involved in energy metabolism, oxidative stress, intracellular Ca^2+^ ([Ca^2+^]_i_) homeostasis [3] and cell death [4]. Under stress conditions, such as ischaemia–reperfusion injury (IRI), mitochondrial damage disrupts ATP synthesis, leading to [Ca^2+^]_i_ overload, and generates large amounts of reactive oxygen species (ROS), which cause oxidative damage to phospholipids, proteins, and ribonucleic acids. Oxidative damage dysregulates mitochondrial morphology and function, and causes further tissue impairment [5]. In addition, ROS bursts amplify the inflammatory response by inducing the release of pro-inflammatory factors and chemokines, leading to microcirculatory ischaemia and vascular endothelial dysfunction [6, 7]. RIRI pathogenesis is complex and involves interactions between oxidative stress, inflammatory response, [Ca^2+^]_i_ overload, apoptosis, and mitochondrial dysfunction [8]. Recent extensive research has shown that mitochondrial biogenesis, energy metabolism, and dynamics are affected during RIRI. Here, we review the latest relevant studies on this topic and describe how disturbances in the morphological and functional characteristics of mitochondria contribute to RIRI.

## Morphology and function of mitochondria

**Fig. 1:**
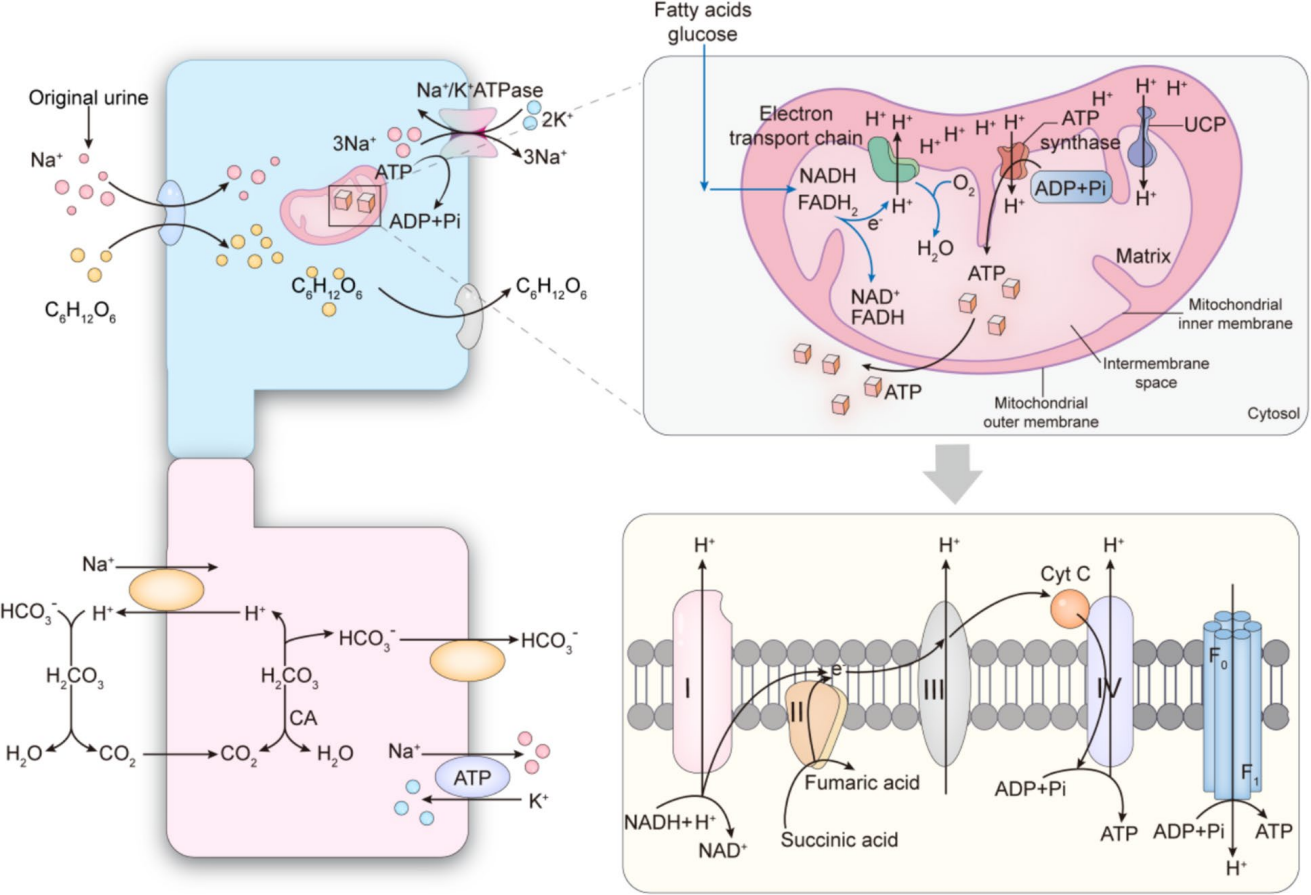
Inverse concentration gradient transport of Na^+^ via the sodium pump in the proximal tubule of the kidney. Primary active transport refers to the transport mode against the concentration gradient directly powered by ATP. The sodium pump is a special protein embedded in the lipid membrane bilayer, possessing ATPase activity and capable of decomposing ATP to release energy for the active transport of Na^+^ and K^+^. The processes of pumping Na^+^ out and pumping K^+^ in are coupled. Under physiological conditions, for each molecule of ATP decomposed, three Na^+^ ions are pumped out of the cell, while two K^+^ ions are pumped back into the cell

The mitochondria are divided from outside to inside into four functional regions: the outer mitochondrial membrane (OMM), mitochondrial membrane gap, inner mitochondrial membrane (IMM), and mitochondrial matrix. Mitochondria produce ATP, appropriate ROS, buffer cytoplasmic Ca^2+^ [9], release inflammatory cytokines, and regulate cellular energy use, signalling, and apoptosis [10]. Large amounts of ATP are required for the filtration and reabsorption in the kidney [11]. Tubular epithelial cells (TECs) depend on fatty acid β-oxidation and mitochondrial oxidative phosphorylation to maintain fluid and solute transport in the body [12]. TECs are particularly sensitive to ischaemia and hypoxia-mediated oxidative stress injuries owing to their high oxygen consumption and mitochondria-rich nature. IRI is accompanied by alterations of mitochondrial morphology, including swelling, fragmentation, disruption of membrane integrity, and fracture or absence of mitochondrial cristae [13]. Mitochondrial dysmorphology causes mitochondrial dysfunction, manifested mainly as reduced ATP production, increased ROS levels, and release of pro-apoptotic factors [6]. All of these phenomena disrupt renal function [14]. Ultrastructural analysis showed that during ischaemia, mitochondrial fragmentation occurs within 15 min after reperfusion in a mouse model of RIRI, ultimately leading to mitochondrial swelling and destruction of tightly packed cristae [15]. Pabla et al. [16] identified a rapid decline in mitochondrial membrane potential in renal proximal tubule mitochondria in a mouse model of RIRI as compared with that in sham-operated mice, which resulted in mitochondrial fragmentation. The maintenance of normal mitochondrial morphology and function is a crucial factor in RIRI control (Fig. 1).

## Mitochondrial biogenesis and RIRI

Mitochondrial biogenesis, i.e., the process of generating new functional mitochondria, increases ATP production and sustains normal mitochondrial homeostasis [17], which represents a complex process involving the synthesis of IMM and OMM, synthesis and import of nuclear-encoded mitochondrial proteins, and replication of mitochondrial DNA (mtDNA) [18]. Peroxisome proliferator-activated receptor gamma-coactivator 1-alpha (PGC-1α) is the most crucial transcriptional regulator of mitochondrial biogenesis [19]. PGC-1α interacts with many transcription factors to facilitate the transcription of mitochondrial proteins and mtDNA replication, and regulates nearly all processes associated with mitochondrial biogenesis [20–22]. A lower expression of the PGC-1α gene has been demonstrated in several different animal models of AKI [23]. RIRI downregulates the expression of PGC-1α and relevant transcription factors, likely through the action of inflammatory cytokines such as TNF-α and IL-1β, which are upregulated during ischemia and reperfusion. These cytokines activate signaling pathways that suppress PGC-1α transcription, contributing to impaired mitochondrial biogenesis and function. Conversely, activation of PGC-1α promotes the expression of enzymes involved in both the de novo nicotinamide adenine dinucleotide (NAD^+^) biosynthesis pathway, which starts with tryptophan, and the salvage pathway for NAD^+^. These pathways are crucial for maintaining mitochondrial function and energy production. By enhancing NAD^+^ levels, PGC-1α supports mitochondrial homeostasis, facilitating recovery from AKI and restoring renal function [24].

## Mitochondrial dynamics and energy metabolism in RIRI

Mitochondrial dynamics encompass mitochondrial fission, fusion, and autophagy, maintained in equilibrium under diverse metabolic conditions [17]. During RIRI, the disruption of mitochondrial dynamics, excessive mitochondrial fission resulting in the accumulation of mitochondrial fragments, overactivation of apoptotic pathways, and formation of ROS and cytochrome C are important factors that exacerbate renal tubular injury [25, 26].

### Mitochondrial fission and fusion

**Fig. 2:**
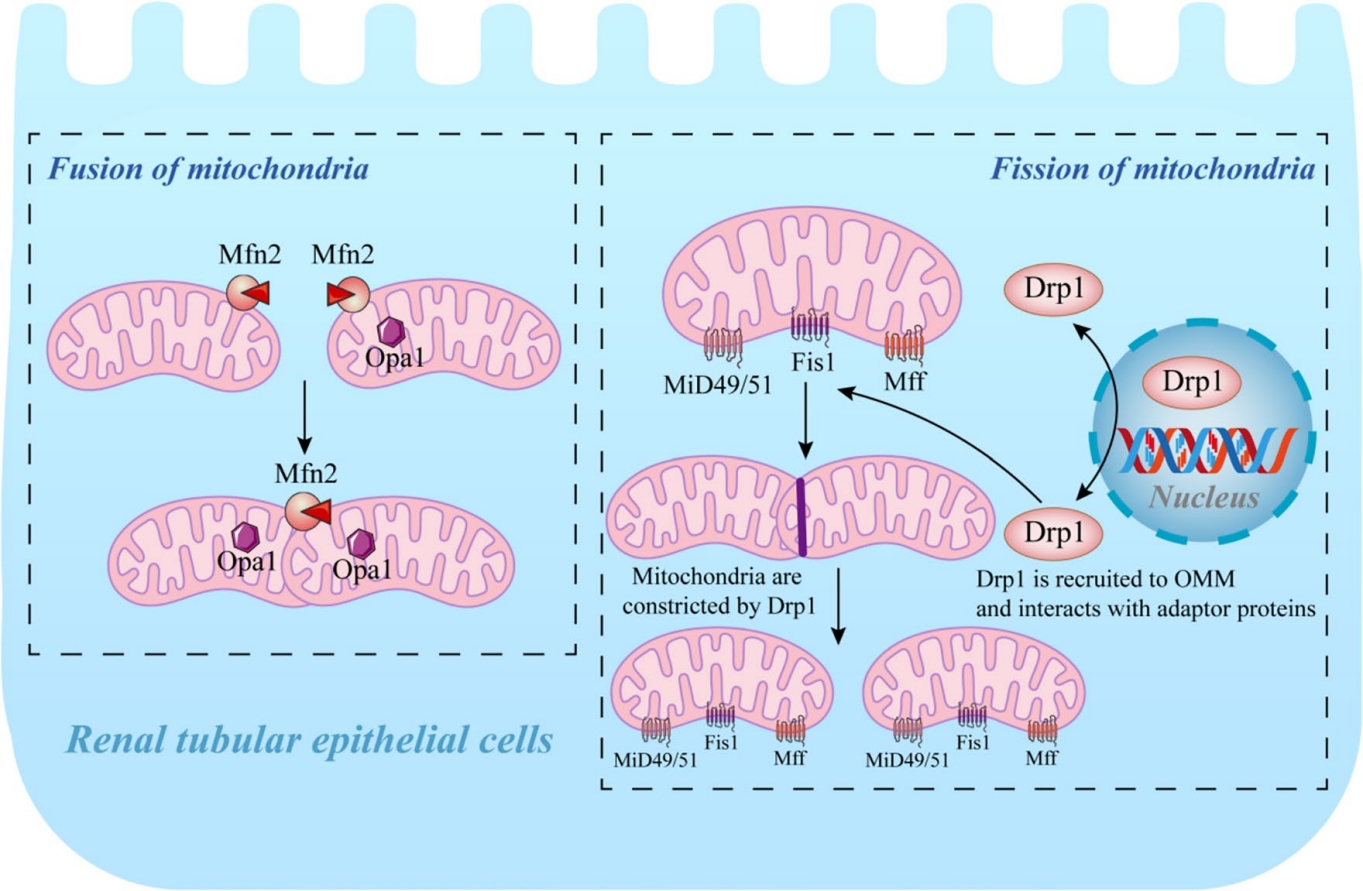
Mitochondrial fission and fusion. Mitochondrial fission and fusion are two interrelated dynamic mitochondrial processes. Mitochondrial fission refers to the fragmentation of mitochondria, which is regulated by its associated proteins, such as DRP1. Mitochondrial fusion is a merger of two adjacent mitochondria, including the OMM and IMM, to form a fibrillar extension and a network of structured mitochondria. Mitochondrial fusion occurrence is regulated by MFN1, MFN2 (located in the OMM), and OPA1 (located in the IMM)

Mitochondrial fission and fusion are important for maintaining mitochondrial homeostasis. Persistent fission and elongation are indispensable for maintaining mitochondrial morphology, energy production, physiological function, cellular homeostasis, and viability [27]. Fusion and fission events are mediated by highly conserved dynamin-related proteins (DRPs) [28]. Dynamin-related protein 1 (DRP1) plays a pivotal role in mediating mitochondrial fission and fusion (Fig. 2) [29]. Mitochondrial damage resulting from excessive mitochondrial fission following renal ischaemia is responsible for renal tubular injury and cell death [30]. Ser637 and Ser616 are the predominant phosphorylation sites of DRP1 [27]. Ser616 phosphorylation promotes mitochondrial fission during mitosis, whereas Ser637 phosphorylation leads to mitochondrial elongation and deformation, which are implicated in apoptosis induction [31]. DRP1 overexpression is the primary cause of I/R-induced mitochondrial fragmentation in proximal renal tubular cells [32], and inhibition of DRP1 expression mitigates excessive proximal tubular mitochondrial fragmentation and alleviates RIRI [33]. Mitochondrial fusion involves the fusion of the OMM and IMM. A reverse parallel dimer, formed between neighbouring mitochondria via the heptapeptide repeat structural domain 2 (HR2) at the C-terminal end of MFN1/2 proteins located in the OMM, is hydrolysed by the N-terminal guanosine triphosphatase to facilitate fusion of the OMM [34]. OPA1, located in the IMM, participates in mitochondrial cristae remodelling in response to oxidative stress or mitochondrial damage and mediates IMM fusion [35]. Sequencing data suggest that IRI induces Fission 1 protein overexpression and downregulates the expression of Optic atrophy 1 (OPA1) and Mitofusin 2 (MFN2) [36]. Sirtuin 3 (SIRT3) functions as a mitochondrial protector that prevents TEC necrosis under stress by enhancing mitochondrial fusion and activating the ERK–Opa1 signalling pathway [37]. Additionally, Du et al. [38] showed that the inhibition of TRIM35-mediated ubiquitination of TIGAR enhanced mitochondrial fusion, prevented mitochondrial dysfunction, and decreased ROS production, thereby mitigating RIRI.

### Mitochondrial autophagy

Mitochondrial autophagy is a quality control mechanism that selectively eliminates damaged and dysfunctional mitochondria to achieve an adaptive response to various stress injuries [39]. Renal hypoxia, inflammation, oxidative damage, and other pathological processes induced by RIRI trigger mitochondrial autophagy [40], which inhibits the excessive production of ROS and release of mitochondrial pro-apoptotic factors, thereby suppressing pathological inflammatory responses [41].

**Fig. 3:**
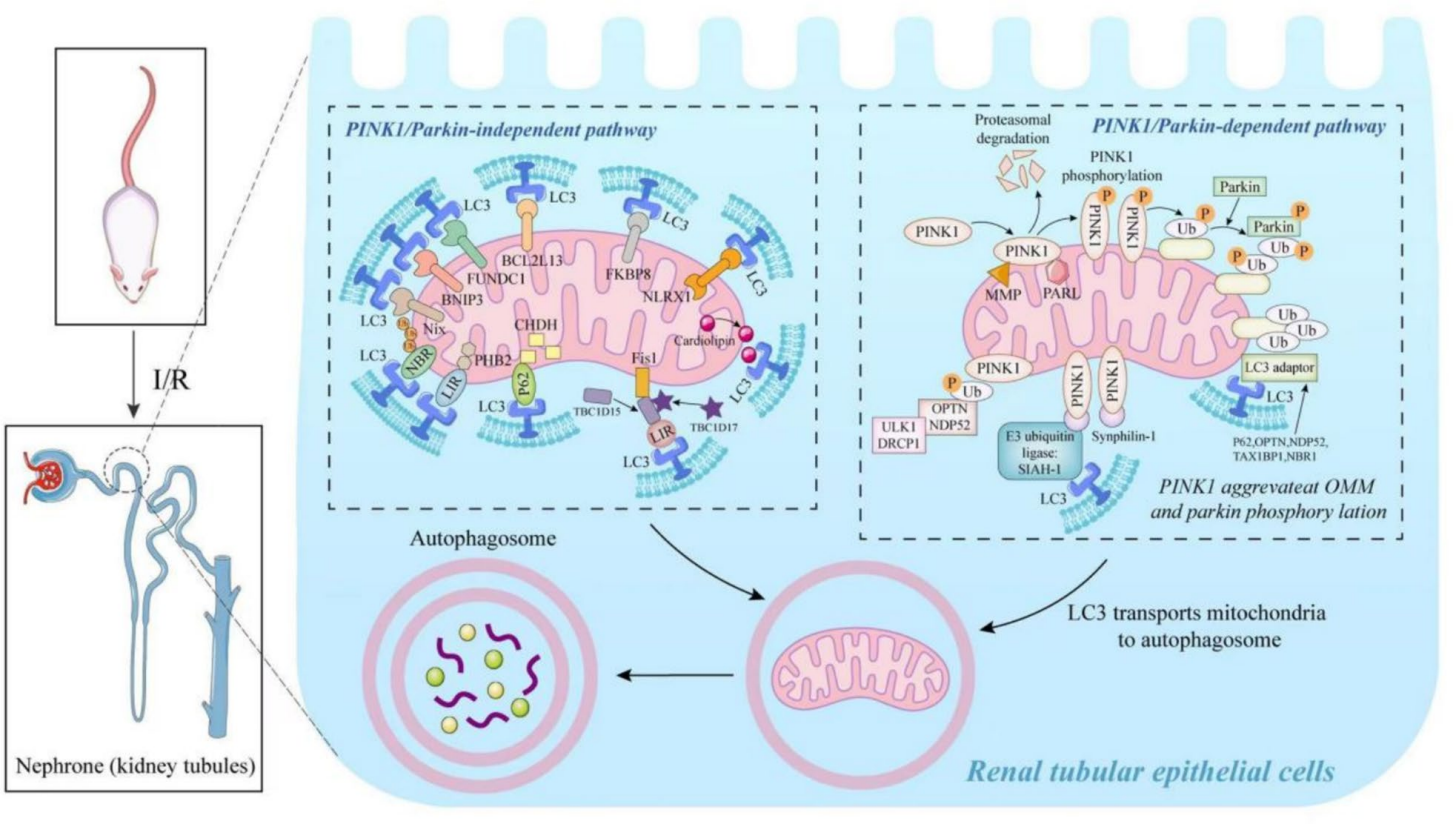
Mitochondrial autophagy in RIRI. During RIRI, mitochondrial damage reduces mitochondrial membrane potential. PINK1 accumulates on the OMM and undergoes autophosphorylation, activating parkin to ubiquitinate proteins on the OMM. LC3 transports ubiquitinated mitochondria to the autophagosome, promoting mitochondrial autophagy. Receptor proteins such as BNIP3, NIX, and FUNDC1 are situated on the OMM and bind to LC3 during the onset of RIRI, inducing mitochondrial autophagy

Mitochondrial autophagy relies on the ubiquitin-dependent and non-ubiquitin-dependent pathways. The ubiquitin-dependent pathway is mainly regulated by the interaction of PTEN-induced kinase 1 (PINK1) and Parkin (PARK2) to form mitochondrial ubiquitin chains, thereby triggering mitochondrial autophagy [42]. The non-ubiquitin-dependent pathway is mainly mediated by autophagy receptors such as BNIP3-like (NIX), Bcl-2/adenovirus E1B 19-kDa interacting protein 3 (BNIP3), and FUN14 domain-containing protein 1 (FUNDC1), which bind directly to microtubule-associated protein 1A/1B-light chain 3 (LC3) to initiate mitochondrial autophagy [43]. It has been shown that RIRI induces mitophagy (Fig. 3) [44].

### PINK1/parkin pathway and RIRI

PINK1, a mitochondrial serine/threonine protein kinase encoded by *PARK6*, is widely expressed in organs that consume a lot of energy, such as the heart, kidney, and brain [45]. Parkin, also known as PARK2, is an extremely versatile E3 ubiquitin ligase. The ubiquitin-mediated mitochondrial autophagy relies on the interaction between PINK1 and Parkin [46]. Usually, PINK1 is predominantly located in the OMM and promptly degraded by the ubiquitin–proteasome system after being constantly recruited to the IMM under physiological circumstances [47]. When the mitochondria are impaired, the mitochondrial membrane potential is diminished, which hinders the translocation of PINK1 to the IMM and results in its accumulation and autophosphorylation on the OMM [48]. Parkin acquires ubiquitin ligase activity [49] and ubiquitinates target proteins on the OMM [50]. The ubiquitinated mitochondria become selectively translocated to autophagosomes through LC3 for eventual degradation by lysosomes [51].

Multiple studies showed that mitochondrial autophagy is protective against RIRI [40, 52]. The deletion of proteins such as PINK1, PARK2, and BINP3 impedes mitochondrial autophagy and exacerbates ROS accumulation, which, in turn, results in mitochondrial accumulation and TEC damage, ultimately aggravating renal injury [44, 53]. Tang et al. [44] showed that mitochondrial autophagy was induced in renal proximal tubular cells in *Pink1* and *Prkn* single- and double-knockout mouse models of ischaemic AKI ex vivo and in vivo, confirming that mitochondrial autophagy mediated by the PINK1–PARK2 pathway plays a crucial role in mitochondrial quality control, proximal tubular cell survival, and renal function during AKI. Livingston et al. [54] proposed that ischaemic preconditioning might prevent renal proximal tubular cell apoptosis and mitigate RIRI by inducing mitochondrial autophagy through the activation of PINK1 to reduce ROS production.

### Receptor-mediated mitochondrial autophagy and RIRI

BNIP3 and NIX (also known as BNIP3L) are homologous pro-apoptotic factors that belong to the Bcl2 family [55]. BNIP3, strongly expressed in various human cells, is directly regulated by hypoxia-inducible factor-1 (HIF-1) [56] and has a high affinity for LC3 [57], a key player in mitochondrial autophagy [58]. BNIP3 expression was elevated in renal tubules after RIRI in mice [59]. Moreover, BNIP3 overexpression alleviated renal injury and enhanced mitochondrial autophagy in mice with HIF-1α knockout in renal proximal tubular cells that underwent RIRI. In vitro investigations showed that HIF-1α knockdown significantly attenuated mitochondrial autophagy induced by hypoxia/reoxygenation (H/R), exacerbated H/R-induced apoptosis, and increased ROS production, whereas BNIP3 overexpression effectively reversed this outcome. Similarly, in vivo experiments showed that HIF-1α knockdown in renal proximal tubular cells markedly inhibited mitochondrial autophagy induced by I/R, aggravated I/R-induced renal tubular apoptosis and kidney injury, whereas BNIP3 overexpression significantly alleviated the reduction of mitochondrial autophagy. Thus, mitochondrial autophagy, mediated by HIF-1α and BNIP3, prevents AKI by reducing renal tubular apoptosis and ROS production. Therefore, HIF-1α plays a protective role in RIRI, and BNIP3 acts as a direct downstream regulator of HIF-1α under hypoxia [60].

NIX functions as a receptor protein that interacts with members of the Atg8 family via LIR to induce mitochondrial autophagy [61]. NIX overexpression reduces the mitochondrial membrane potential [62, 63], modulates the translocation of parkin to the mitochondria, and activates parkin–ubiquitin–p62-mediated mitochondrial autophagy [64, 65]. Xu et al. [66] demonstrated that NIX-mediated autophagy ameliorated proteinuria-induced renal tubular cell apoptosis and renal injury. Pan et al. [67] showed that enhancing NIX-dependent mitochondrial autophagy in platelets and limiting the overactivation of inflammatory vesicles alleviated AKI.

The FUNDC1 protein is located in the OMM and directly interacts with LC3 to induce mitochondrial autophagy under hypoxic conditions [68]. FUNDC1 is a downstream regulator of HIF-1α during mitochondrial autophagy of renal proximal tubular cells, and the HIF-1α/FUNDC1 signalling pathway mediates the increase in filamentous lysosome formation induced by H/R, which reduces apoptosis and ROS production, thereby protecting against RIRI [67].

Nevertheless, numerous findings also indicated the dual nature of mitochondrial autophagy in RIRI [44, 53], although its precise mechanisms remain incompletely elucidated. Decuypere et al. [69] found that during the 20–40 min of renal ischemia, mitochondrial autophagy exerted a protective effect on the kidneys; however, after 40–60 min of ischemia, excessive activation of autophagy became detrimental. Specifically, during ischemia, moderate mitochondrial autophagy removes damaged mitochondria, reducing ROS accumulation, mitochondrial DNA release, and pro-apoptotic factor production, thus effectively alleviating oxidative damage. This protective effect is likely mediated by the PINK1/Parkin signaling pathway [70]. However, the activation of mitochondrial autophagy during reperfusion after the initial hypoxic state might exacerbate cellular damage [71]. The specific mechanisms responsible for this dual role of autophagy in mitochondria remain ambiguous [72] and require further investigation. The expression and activity of autophagy receptors, such as BNIP3, NIX, and FUNDC1, at different stages may play a crucial role in determining whether mitochondrial autophagy has a protective or damaging effect. Therefore, how to precisely regulate the levels and timing of autophagy at different stages remains a critical focus for future research. Exploring the use of small molecules or gene-editing technologies to fine-tune mitochondrial autophagy will help prevent damage caused by excessive autophagy while maximizing its protective effects.

## Treating RIRI by regulating mitochondria

### Pharmaceutical agents

Metformin, widely used for type 2 diabetes, also exhibits anti-oxidative, anti-inflammatory, and mitochondrial-protective effects. It mitigates RIRI by activating the AMPK-regulated SENP1–SIRT3 pathway, improving proximal tubular function and reducing renal inflammation and fibrosis [73]. Empagliflozin is a sodium-glucose cotransporter 2 (SGLT2) inhibitor used in diabetes management. Its role in mitochondrial regulation and anti-inflammatory effects has been increasingly studied in the context of AKI. Empagliflozin alleviated RIRI by mitigating inflammation and facilitating mitochondrial fusion through the AMPK–OPA1 pathway [74]. Sufentanil is an opioid analgesic commonly used for anesthesia and pain management. In a cellular model of RIRI, Liu et al. [75] demonstrated that sufentanil ameliorated RIRI by attenuating H/R-induced apoptosis, mitochondrial dysfunction, oxidative stress, inflammation, and activation of the PI3K/AKT/FOX01 pathway. Mitochondria generate ROS in cells, so promoting ROS scavenging, inhibiting ROS production, and safeguarding mitochondria from oxidative damage constitute crucial therapeutic strategies for ameliorating RIRI. In animal experiments, it has been shown that mitochondria-targeted antioxidants MitoQ and SkQR1 lowered mitochondrial ROS levels and protected mitochondria from oxidative damage, thus attenuating RIRI [76, 77]. As an oxygen radical scavenger clinically used for stroke, edaravone reduces oxidative stress by inhibiting JAK2 and STAT1/STAT3 phosphorylation, which in turn decreases pro-apoptotic signals and improves mitochondrial function. This helps preserve mitochondrial integrity and promotes cell survival during I/R injury [78]. Roxadustat, an oral Hypoxia-inducible factor prolyl hydroxylase inhibitor (HIF-PHI), is primarily used for treating anemia in chronic kidney disease. Beyond its hematopoietic effects, it enhances HIF-1α-mediated mitophagy, which plays a protective role in RIRI. Zhang et al. [79] demonstrated that roxadustat upregulates HIF-1α, activating the FUNDC1 signaling pathway, which promotes mitophagy, reduces ROS accumulation, and inhibits renal tubular apoptosis. In a rat RIRI model, roxadustat treatment increased mitophagy and reduced renal injury, suggesting its potential as a therapeutic agent for RIRI-induced AKI.

NADPH oxidase 4 (NOX4), mainly located in the mitochondria [80], is upregulated by RIRI [81]. A recent study by Schiffer et al. [82] suggested that NOX4 inhibitor GLX7013114 can preserve mitochondrial function during reperfusion by reducing ROS production, enhancing mitochondrial activity, and promoting protective signaling pathways like nuclear factor erythroid 2-related factor 2 (Nrf2). These effects help reduce apoptosis and improve renal function, offering potential therapeutic benefits for AKI patients. Surprisingly, melatonin, which regulates mitochondrial dynamics and autophagy mainly through AMPK/DRP1, is also protective in RIRI by reducing mitochondrial fission, promoting fusion, and attenuating excessive autophagy [83]. The endogenously derived gaseous transmitter hydrogen sulfide (H_2_S) has been recently shown to alleviate hypoxic tissue impairment. During kidney transplantation, H_2_S mitigates RIRI by stimulating ATP production, modulating Ca^2+^ channels, and preserving mitochondrial membrane integrity, predominantly by providing electrons to the electron respiratory chain. Furthermore, H_2_S targets mitochondria with up to a 1,000-fold higher potency during cold renal H/R injury in vitro [84]. In addition, P110, a novel DRP1-selective peptide inhibitor, protects against RIRI by inhibiting mitochondrial fragmentation and ROS production, which improves the mitochondrial membrane potential and mitochondrial integrity [85].

Table 1 summarizes various pharmaceutical agents that have been shown to modulate mitochondrial function in order to alleviate RIRI. The table outlines the mechanisms of action of several drugs, including their effects on key mitochondrial processes such as oxidative stress, mitochondrial dynamics, and mitochondrial biogenesis.

**Table 1:** Pharmacological modulation of mitochondria to alleviate RIRI

Drug	Classification	Mechanism of action	Reference
Metformin	Biguanide	AMPK↑, SIRT3↓, ROS↓, ATP↑, PTEC apoptosis↓	[73]
Empagliflozin	Sodium-glucose cotransporter protein 2 inhibitor	OPA1↑, AMPK↑, mitochondrial fission↓, mitochondrial fusion↑, maintaining mitochondrial homeostasis	[74]
Sufentanil	Opiates narcotics	PI3K/AKT/FOX01 pathway↑, PINK1↓, amelioration of mitochondrial membrane potential (MMP) dysfunction, oxidative stress, and inflammation	[75]
MitoQ	Antioxidant	ROS↓, mitochondrial oxidative damage↓	[76]
SkQR1	Antioxidant	ROS↓, mitochondrial oxidative damage↓	[77]
Edaravone	Oxygen radical scavenger	JAK2↓, STAT3↓, Bcl-2↑, Bax↓, MMP↑, mitochondrial ultrastructural damage↓, mtROS↓	[78]
Roxarestat	Antianaemic drug	HIF-1α/FUNDC1↓, renal tubular apoptosis↓, mtROS↓, mitochondrial autophagy↓	[79]
GLX7013114	NOX4 inhibitor	NOX4↓, mtROS↓, PGC-1α↑, mitochondrial biogenesis↑	[82]
Melatonin	Hormone	maintaining the balance between mitochondrial fission and autophagy	[83]
H_2_S	Gaseous transmitter	ETC↑, regulation of Ca^2+^ channels, mitochondrial membrane integrity↑	[84]
P110	DRP1 selective peptide inhibitor	mitochondrial hypersegmentation↓, mtROS↓, MMP↑, mitochondrial membrane integrity↑	[85]

*AMPK* adenosine monophosphate activated protein kinase, *ATP* adenosine triphosphate, *AKT* Akt kinase, *Bax* Bcl-2 antagonist X, *Bcl-2* B-cell lymphoma 2, *ETC* electron transport chain, *FUNDC1*, FUN14 domain containing 1, *HIF-1α* hypoxia-inducible factor 1-alpha, *JAK2* Janus kinase 2, *MMP* matrix metalloproteinase, *NOX4* NADPH oxidase 4, *OPA1* optic atrophy 1 (OPA1 mitochondrial dynamin like GTPase), *PGC-1α* peroxisome proliferator-activated receptor-γ coactivator 1 alpha, *PTEC* proximal tubular epithelial cell, *ROS* reactive oxygen species, *SIRT3* sirtuin 3, *STAT3* signal transducer and activator of transcription 3

### Chinese medicine ingredients

In recent years, numerous studies have revealed that Chinese herbs and their extracts provide effective ways of multi-target and multi-pathway regulation of mitochondria to ameliorate RIRI. Wu et al. [86] demonstrated that chrysin, a biologically active constituent of the flowers of *Abelmoschus manihot* (L.) Medik. protected against RIRI by modulating mitochondrial fission, oxidative stress, and apoptosis mediated by the OMA1/OPA1 signalling pathway. Zhong et al. [87] showed that *Ganoderma lucidum* polysaccharide peptides scavenged ROS, alleviated oxidative stress, and reduced apoptosis induced by mitochondrial and endoplasmic reticulum stress in vitro and in vivo. Ligustilide is a natural compound extracted from *Ligusticum chuanxiong* and *Angelica sinensis*. Xia et al. demonstrated in vitro and in vivo that ligustilide mitigated oxidative stress during RIRI by maintaining mitochondrial homeostasis through the targeted upregulation of SIRT3 [88]. Resveratrol protects kidneys via the SIRT3–FOXO3a pathway, and its mechanism of action encompasses not only the regulation of the oxidation–reduction balance and inhibition of cell apoptosis but also enhancement of PGC-1α expression to facilitate mitochondrial generation [89]. Qi et al. [90] showed that loureirin C, an extract of *Dracaena cochinchinensis*, inhibited renal oxidative stress and ferroptosis by activating Nrf2 and its downstream target genes, which in turn enhanced mitochondrial function and renoprotection during RIRI. Lacin belongs to the class of flavonoids that maintain TEC activity, improve ATP production in renal tissues, reduce ROS release, and enhance mitochondrial function, thereby attenuating RIRI consequences [91]. Quercetin is a flavonoid antioxidant that affects mitochondria. Quercetin reduced apoptosis in intravascular smooth muscle cells by blocking oxidative stress and mitochondrial fission [92] and attenuated RIRI [93].

Table 2 outlines the key components of traditional Chinese medicine and their mechanisms of action in regulating mitochondrial function to alleviate RIRI. These ingredients target multiple mitochondrial processes, such as oxidative stress, mitochondrial dynamics, and apoptosis.

**Table 2:** Regulation of mitochondria by components of traditional Chinese medicine preparations to relieve RIRI

Ingredient	Mechanism of action	Reference
Chrysin	Modulation of the OMA1–OPA1 pathway, attenuation of mitochondrial fission, reduction of oxidative stress and apoptosis	[86]
*Ganoderma lucidum* polysaccharide peptides	Scavenging of ROS, reduction of oxidative stress, reduction of apoptosis induced by mitochondrial and endoplasmic reticulum stress	[87]
Ligustilide	Upregulation of SIRT3 maintains mitochondrial homeostasis to attenuate oxidative stress during RIRI	[88]
Resveratrol	Modulation of the SIRT3–FOXO3a pathway, increase in PGC-1α expression promoting mitochondrion generation	[89]
Loureirin C	Activation of Nrf2 and its downstream target genes, inhibition of oxidative stress and ferroptosis, improvement of mitochondrial function	[90]
Lacin	Maintenance of the ETC activity, increased ATP production, reduced ROS generation	[91]
Quercetin	Inhibition of oxidative stress, suppression of mitochondrial fragmentation, attenuation of cell apoptosis	[92]

*ATP* adenosine triphosphate, *ETC* electron transport chain, *FOXO3* forkhead box O3, *Nrf2* Nuclear factor erythroid 2-related factor 2, *OMA1* overlapping proteolytic activity with m-AAA protease 1, *OPA1* optic atrophy 1 (OPA1 mitochondrial dynamin like GTPase), *PGC-1α* peroxisome proliferator-activated receptor-γ coactivator 1 alpha, *ROS* reactive oxygen species, *SIRT3* sirtuin 3

### Potential therapeutic targets

Administration of mesenchymal stromal cells (MSCs) repairs cellular damage in AKI via endocrine or paracrine mechanisms [94]. Extracellular vesicles derived from MSCs carry a variety of biologically active molecules, such as RNA, and represent key tools for intercellular communication [95]. Gu et al. showed that human umbilical cord Wharton’s jelly MSC-derived extracellular vesicles upregulated miR-30b, miR-30c, and miR-30d in renal proximal tubular cells, alleviated DRP1 activation and mitochondrial fragmentation, thereby exerting an anti-apoptotic effect and protecting against RIRI [96]. Activation of the 5-hydroxytryptamine 1F receptor alleviated pathological mitochondrial damage through the upregulation of PGC-1α expression and facilitation of mitochondrial biogenesis, which presents a potential therapeutic approach for AKI [97]. During RIRI, mitochondrial damage is partially manifested by elevated levels of mtROS and substantial mtDNA depletion [98]. TFAM **(**Mitochondrial Transcription Factor A**)** is a key regulator of mitochondrial DNA transcription and replication. Zhao et al. demonstrated that mtROS decreased TFAM content in TECs by inhibiting TFAM transcription and promoting Lon-mediated TFAM degradation. TFAM deficiency induced depletion of mtDNA and mitochondrial respiration defects in TECs during RIRI, which further reduced mtDNA synthesis and mitochondrial biogenesis. Hence, TFAM deficiency may be an important therapeutic target in RIRI [99]. Another study showed that stanniocalcin-1, located in the IMM, regulates AMPK, UCP2 (uncoupling protein 2), and SIRT3 in the kidney and protects against RIRI by activating AMPK [100].AMPK is a key regulator of energy production in most cell types. Ma et al. [102] reported that I/R triggered ceramide production and activation of the protein phosphatase PP2A, which dephosphorylates Thr172-AMPKα, in experiments in vitro and in vivo. The reduced activity of AMPK inhibits the serine/threonine kinase Unc-51 like autophagy-activating kinase 1-mediated autophagy, which hampers the clearance of dysfunctional mitochondria. Therefore, AMPKα activator C24 was used to target the PP2A–AMPK axis to restore fatty acid oxidation and reduce proximal renal tubular cell apoptosis during RIRI by antagonizing PP2A dephosphorylation and promoting mitochondrial autophagy.

Bax inhibitor-1 (BI1) conveys anti-apoptotic signals within mitochondria, whereas prohibitin 2 (PHB2) preserves mitochondrial morphology and function. Wang et al. observed lower levels of BI1 in the urine and plasma of patients with AKI, compared to those in healthy controls, and concluded that BI1 expression negatively correlated with renal function. Furthermore, BI1 significantly affected the renal tubular function by regulating the mitochondrial localisation of PHB2 in an AKI mouse model. BI1 maintains mitochondrial genetic integrity, mitigates mitochondrial oxidative stress, promotes mitochondrial respiration, inhibits mitochondrial hyperfission, enhances mitochondrial autophagy, and suppresses mitochondrial apoptosis. Thus, the direct BI1–PHB2 interaction may constitute a crucial mitochondrial apoptotic regulatory target in the management of AKI [101]. Renal TECs generate ATP primarily by fatty acid oxidation. Feng et al. showed that the expression of mammalian STE20-like kinase 1 (MST1) was augmented in response to RIRI in vivo, and that elevated levels of MST1 positively correlated with renal insufficiency and augmented apoptosis in TECs. In addition, genetic ablation of MST1 conferred a survival advantage upon renal TECs by inhibiting mitochondrial fission through the reactivation of the AMPK–YAP–OPA1 signalling pathway [103]. A crosstalk exists between connexin 32 and the mitochondrial apoptotic signalling pathway. Furthermore, IR-induced AKI was alleviated through the inhibition of connexin 32 function, reduction of ROS generation, and suppression of connexin 32 downstream NF-κB/p53/PUMA-mediated mitochondrial apoptotic signalling pathway [104]. Irisin, a myokine secreted by skeletal muscles during exercise, is a cleavage product of fibronectin type III domain-containing protein 5. Cui et al. [105]. showed that irisin promoted mitochondrial integrity and function by up-regulating mitochondrial autophagy-related proteins LC3, PINK1, and PARK2, and down-regulating reaction substrate proteins chelator 1 (p62), mitochondrial extramembranous translocase 20 (TOM20), and triton endomembrane translocase 23 (TIM23) to prevent RIRI. Tran et al. hypothesised that PGC1α-dependent NAD biosynthesis could link oxidative metabolism with renal protection. In their study, they observed that subsequent to renal ischaemia, PGC1α^−/−^ mice showed a local deficit of the NAD precursor nicotinamide, substantial fat accumulation, and inability to re-establish normal function. Treatment with exogenous nicotinamide augmented local NAD levels, fat accumulation, and improved renal function in PGC1α^−/−^ mice post-ischaemia. Nicotinamide and NAD may also provide novel perspectives for the treatment of IRI-AKI [106].

Table 3 summarizes potential therapeutic targets and their mechanisms for mitochondrial modulation in the treatment of RIRI. The table highlights various mitochondrial dysfunction pathways and associated targets that can be regulated to alleviate RIRI.

**Table 3:** Potential therapeutic targets and mechanisms of mitochondrial modulation to alleviate RIRI

Type of mitochondrial dysfunction	Therapeutic target	Protection mechanism	Laboratory animal	Method of model induction	Reference
Mitochondrial dynamics	hWJMSC-EVs	miR-30b/c/d↑, DRP1↓, mitochondrial fission↓	Male SD rats	Right nephrectomy with 45-min vascular clamping of the left renal pedicle	[96]
Mitochondrial biogenesis	5-Hydroxytryptamine 1F receptor	PGC-1α↑, mitochondrial biogenesis↑	Male C57BL/6NCr mice	Bilateral renal tip vascular clamp closure for 18 min	[97]
Mitochondrial energy metabolism	TFAM	mtROS↑, TFAM↑, mtDNA ↓, mitochondrial energy metabolism↓	Male C57BL/6 mice	Bilateral renal tip vascular clamp closure for 30 min	[99]
	BI1–PHB	mtRNA ↑, oxidative stress↓, ETC↑, mitochondrial autophagy↓, apoptosis ↓	C57BL/6 male mice, BI1 transgenic (BI1TG) male mice on the C57BL/6 background	Bilateral renal tip vascular clamp closure for 30 min	[101]
	AMPK	AMPK activator C24, PP2A↓, Thr172-AMPK dephosphorylation↓, AMPK↑, mitochondrial autophagy↑, FAO↑, Mitochondrial mass↑	Male C57BL/6 J mice	Bilateral renal tip vascular clamp closure for 30 min	[102]
	Mst1	MST1↑, AMPK-YAP pathway↓, OPA1↓, mitochondrial autophagy↓, mitochondrial apoptosis↑	AMPKα1/α2 double knockout male mice	Unilateral renal tip vessel clamp closure in 30 min	[103]
	STC1	AMPK↑, UCP2↑, sirtuin 3↑, ROS↓	STC1 Tg male mice on the C57BL/6 genetic background	Bilateral renal tip vascular clamp closure for 30 min	[100]
	Connexin 32	Connexin 32↓, ROS↓, anti-apoptotic factor PUMA↓, NF-κB/p53/p53↓, mitochondrial apoptosis↓	C57BL/6 male mice CX32^+/−^ male mice on the C57BL/6 background	Bilateral renal tip vascular clamp closure for 45 min	[104]
	Irisin	LC3↑, PINK1↑, PARK2↑, p62↓, TOM20↓, TIM23↓, mitochondrial integrity maintenance	Male C57BL/6 mice	Bilateral renal tip vascular clamp closure for 18 min	[105]
Antioxidant defence systems	Nam	Exogenous Nam, NAD ↑, fat accumulation ↓ β-hydroxybutyrate ↑, PGE 2 ↑, oxidative metabolism ↑, renal function ↑	Male PGC1α^−^/^−^ (progenitor # 008597), Pax8-rtTA (# 007176) and TRE-PGC1α (# 012387) mice	Bilateral renal tip vascular clamp closure for 20 min	[106]

*AMPK* adenosine monophosphate activated protein kinase, *ATP* adenosine triphosphate, *DRP1* dynamin-related protein 1, *LC3* microtubule-associated protein light chain 3, *MST1* macrophage-stimulating protein, *NAD* nicotinamide adenine dinucleotide, *OPA1* optic atrophy 1 (OPA1 mitochondrial dynamin like GTPase), *PARK2* parkin, *Pax8* Paired box gene 8, *PGC-1α* peroxisome proliferator-activated receptor-γ coactivator 1 alpha, *PGE2* prostaglandin E2, *PINK1* PTEN-induced kinase 1, *PUMA* p53-upregulated modulator of apoptosis, *ROS* reactive oxygen species, *STC1* stanniocalcin-1, *TIM23* translocase of inner mitochondrial membrane 23, *TOM20* translocase of outer mitochondrial membrane 20, *TFAM* transcription factor A, mitochondrial, *UCP2* uncoupling protein 2, *YAP1* Yes1 associated transcriptional regulator

## Summary and perspectives

**Fig. 4:**
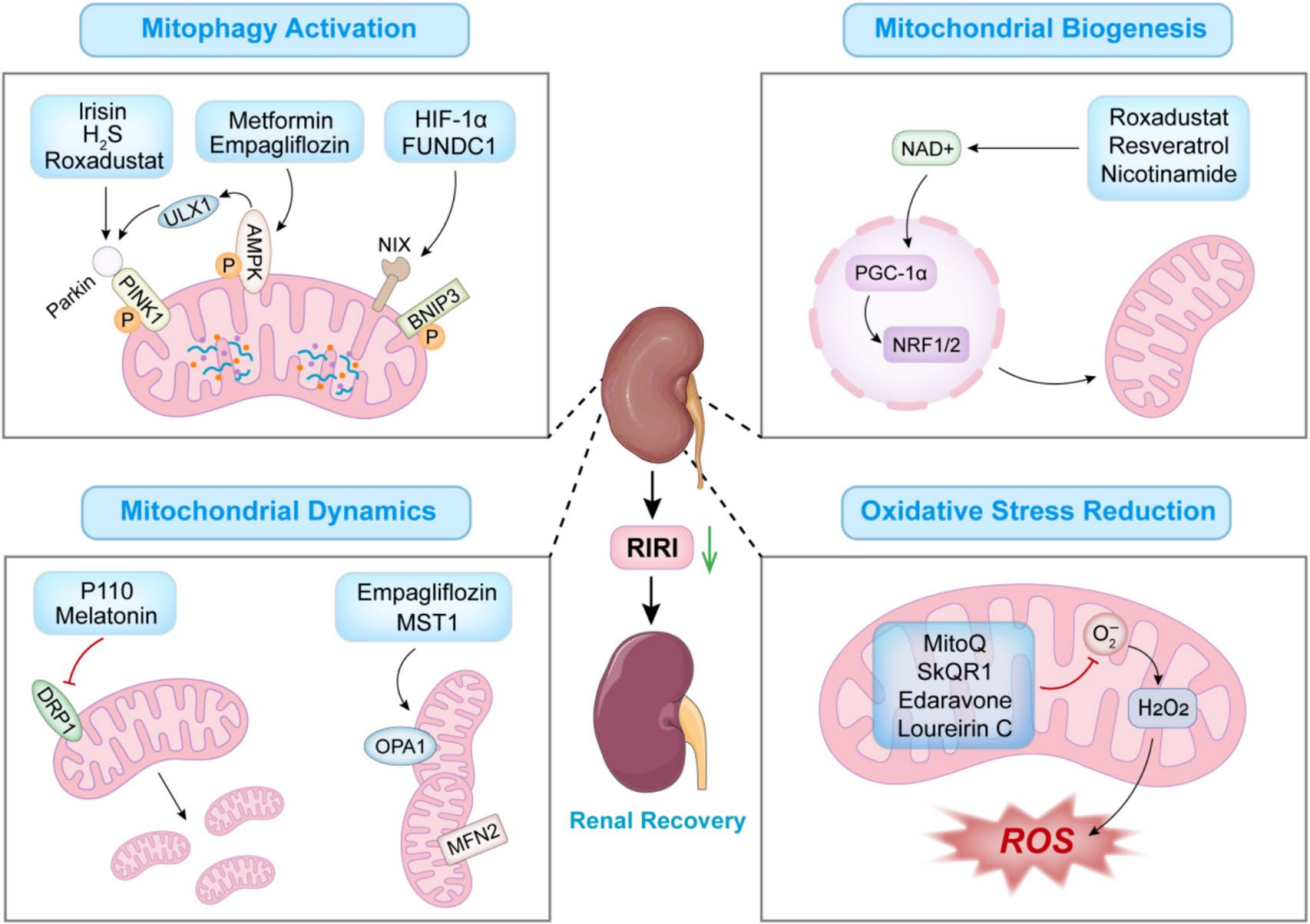
Mitochondrial Pathways in RIRI Treatment. This figure outlines multifactorial therapeutic strategies for RIRI, highlighting synergistic approaches to preserve mitochondrial and renal homeostasis through modulation of mitophagy, mitochondrial biogenesis (PGC-1α/NRF1/2), fission–fusion dynamics (OPA1/Drp1), and oxidative stress (MitoQ, Edaravone). Key pharmacological agents—including AMPK activators (metformin), HIF stabilizers (roxadustat), and redox-targeted compounds—are shown to mitigate RIRI by restoring mitochondrial quality control and redox balance

RIRI is a common clinical cause of AKI. The kidneys have abundant mitochondria, and mitochondrial impairment is an essential pathophysiological characteristic of RIRI. Mitochondrial biogenesis, division and fusion, autophagy, and energy metabolism are closely associated with RIRI, and the preservation of mitochondrial homeostasis is of vital significance for this condition. Furthermore, as Fig. 4 is the final figure in our manuscript, this insertion will not require renumbering of subsequent figures or affect the existing figure sequence. Although effective therapeutic drugs for RIRI are scarce, and some potentially promising targets and drug candidates are only at the preclinical stage, the combined targeting of several proteins based on multiple mechanisms of mitochondrial regulation offers a novel perspective for the treatment of RIRI. Consequently, exploration of mitochondrial processes and in-depth studies of potential mitochondrial targets to rectify the cellular damage caused by RIRI will facilitate the development of effective drugs for clinical application at an early stage and prevention of AKI occurrence.
